# Effect of deep analgosedation vs. intubated general anesthesia on perioperative sedation-related adverse events in older adults undergoing ERCP: protocol for a randomized controlled trial

**DOI:** 10.3389/fmed.2026.1764649

**Published:** 2026-02-26

**Authors:** Xiaocui Lv, Yu Xu, Jianliang Sun, Huacheng Liu, Youjia Yu, Gang Chen

**Affiliations:** 1 Department of Anesthesiology, Sir Run Run Shaw Hospital, Zhejiang University School of Medicine, Hangzhou, China; 2 Department of Anesthesiology, Suzhou Xiangcheng People's Hospital, Suzhou, China; 3 Department of Anesthesiology, Hangzhou First People's Hospital, Westlake University School of Medicine, Hangzhou, China; 4 Department of Anesthesiology, Second Affiliated Hospital of Wenzhou Medical University, Wenzhou, China

**Keywords:** deep analgosedation, ERCP, general anesthesia, NPA-assisted DAS, perioperative sedation-related adverse events

## Abstract

**Background:**

Deep analgosedation (DAS) and general anesthesia (GA) are currently the predominant anesthetic approaches for endoscopic retrograde cholangiopancreatography (ERCP). In contrast to DAS, GA typically requires endotracheal intubation for airway management. To determine the optimal analgosedation strategy, this study is designed to compare the effect of nasopharyngeal airway-assisted DAS (NPA-assisted DAS) vs. endotracheal intubation GA on perioperative sedation-related adverse events (SRAEs) in elderly ERCP patients.

**Methods:**

Patients scheduled for ERCP will be randomly assigned in a 1:1 ratio to NPA-assisted DAS or endotracheal intubation GA. Randomization will be stratified by study center and hypertension status, using permuted blocks of sizes 2 and 4. The major exclusion criterion is severe cardiopulmonary disease. The primary outcome is a composite of SRAEs, including hypoxemia, hypotension, hypertension, laryngospasm, or bronchospasm. Secondary outcomes include escalation of respiratory support, tachycardia, bradycardia, reflux, recovery time, postoperative recovery quality (QoR-15), postoperative nausea and vomiting (PONV), cognitive function, perioperative respiratory failure or acute respiratory distress syndrome (ARDS), patient and endoscopist satisfaction, and length of hospital stay. All analyses will follow a modified intention-to-treat approach.

**Ethics and dissemination:**

Ethics approval was obtained from the Ethics Committee of Sir Run Run Shaw Hospital, Zhejiang University School of Medicine (2025-0780). Written informed consent will be obtained from all participants prior to enrollment. The findings of this trial will be disseminated through publication in a peer-reviewed journal.

**Trial registration number:**

Clinical Trial Registry (NCT07017283).

## 1 Introduction

Endoscopic retrograde cholangiopancreatography (ERCP) is a well-established and widely used minimally invasive technique for the diagnosis and treatment of biliary and pancreatic diseases (1, 2). With the rising prevalence of these disorders, the demand for ERCP continues to increase globally, with an estimated need approaching 600 000 procedures annually in USA alone (3). Despite its minimally invasive nature, ERCP can still cause considerable pain and discomfort (4, 5), making effective analgosedation essential for successful procedure performance and improved patient comfort.

ERCP-related anesthesia is commonly performed outside the operating room, where limited access to emergency equipment may lead anesthesiologists to favor general anesthesia (GA) with endotracheal intubation (6). GA provides a secured airway and may reduce the risk of hypoxemia and procedure interruption. However, GA often requires deeper levels of anesthesia, which may exacerbate hemodynamic instability and contribute to post-extubation hypoxemia in elderly patients with impaired cardiopulmonary reserve (7). Furthermore, GA is associated with intubation-related complications and prolonged recovery time, potentially affecting perioperative outcomes and healthcare efficiency (8).

Deep analgosedation (DAS) is widely adopted in ERCP and provides high patient and endoscopist satisfaction (9, 10). However, because patients under DAS rely on spontaneous breathing without a secured airway, the risk of airway obstruction, hypoxemia, and unplanned conversion to GA remains significant, particularly in elderly patients with reduced cardiopulmonary reserve. Moreover, the prone position commonly required for ERCP makes airway access inherently challenging and may delay timely interventions when obstruction occurs (11). Nasopharyngeal airways (NPAs) can help maintain airway patency and oxygenation during DAS without the need for tracheal intubation, potentially balancing airway protection with advantages such as more stable hemodynamics and faster recovery (12, 13). Given these considerations, NPA-assisted DAS may offer a safer alternative to GA in elderly ERCP patients by reducing sedation-related adverse events.

This study aims to compare nasopharyngeal airway-assisted deep analgosedation with endotracheal intubation general anesthesia in elderly ERCP patients, focusing on perioperative sedation-related adverse events (SRAEs). The results may help identify a safer anesthetic approach for this high-risk population. And the findings will be reported in a future publication following study completion.

## 2 Methods

This protocol adheres to the Standard Protocol Items: Recommendations for Interventional Trials (SPIRIT) guidelines.

### 2.1 Study design and patients

**Figure 1:**
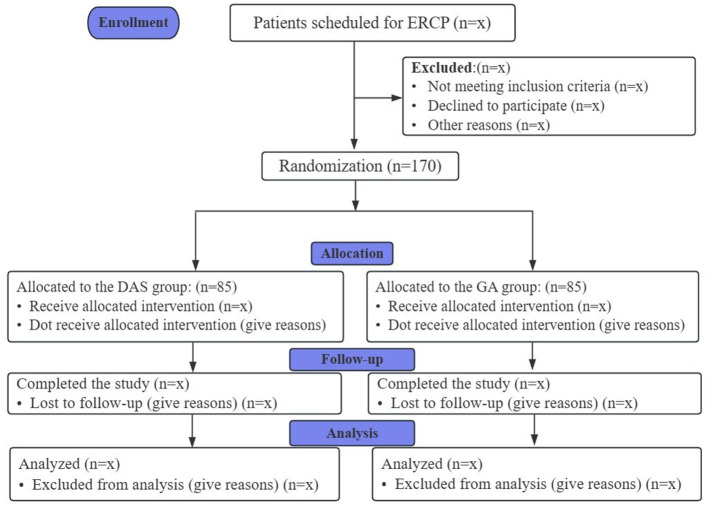
Study flow diagram.

This study is a multicenter, prospective, randomized, open-label, parallel-group clinical trial. It will be conducted at Sir Run Run Shaw Hospital, Zhejiang University School of Medicine, together with two additional tertiary hospitals in Zhejiang Province, with a planned enrollment of 170 patients. Recruitment and follow-up for ERCP procedures will take place from November 2025 to April 2026. The study flow diagram is presented in Figure 1.

#### 2.1.1 Inclusion criteria

- Patients who meet the following criteria will be included.
- Aged 60 and above;
- ASA physical status ≤ III;
- Patients who scheduled for elective therapeutic ERCP [ERCP complexity grading system (14) grades 2–4];
- Willing to provide informed consent (or by legal guardian/witness if applicable).

#### 2.1.2 Exclusion criteria

- The exclusion criteria include.
- Altered gastrointestinal anatomy, delayed gastric emptying, or gastric outlet obstruction;
- Coagulopathy or epistaxis tendency;
- Cardiac diseases (e.g., coronary artery disease, heart failure, arrhythmias);
- Pulmonary diseases (e.g., asthma, COPD);
- Prior hypersensitivity to anesthetic agents;
- Active upper respiratory infection;
- Severe liver and kidney diseases;
- Difficult airway: preoperative anesthesiologist assessment of difficult airway;
- Psychiatric disorders, cognitive impairment, critical illness, or pregnancy.

### 2.2 Primary outcome

The primary outcome is the incidence of a composite endpoint of SRAEs (Intraoperative and during the recovery room period): hypotension (15, 16) (blood pressure decrease exceeding 20% of baseline or mean arterial pressure ≤ 65mmHg, blood pressure was measured again, if the result is still lower than 20%, confirm as hypotension), hypertension (17) (blood pressure increase exceeding 20% of baseline or mean arterial pressure ≥105mmHg, blood pressure was measured again, if the result is still higher than 20%, confirm as hypertension), hypoxemia (SpO_2_ ≤ 90% for >10 s) (18, 19), laryngospasm, bronchospasm (20) (Table 1).

**Table 1:** Schedule of patient enrolment, study interventions and outcome assessment.

**Time point**	**Study period**						
	**Enrollment**	**Allocation**	**Allocation**	**Post-allocation**	**Post-allocation**	**Post-allocation**	**Close-out**
	**Pre-op visit**	**Pre-op**	**Perio-op**	**PACU**	**1 day post-op**	**3 day post-op**	**Discharged**
**Patient enrolment**							
Eligibility criteria	×						
Written informed consent	×						
Demographic data	×						
Baseline characteristics	×						
Randomization/allocation		×					
**Study interventions**							
GA Group			×				
DAS Group			×				
**Outcome assessment**							
**SRAEs**							
Hypotension			×	×			
Hypertension			×	×			
Hypoxemia			×	×			
Laryngospasm			×	×			
Bronchospasm			×	×			
**Secondary outcomes**							
Nasopharyngeal airway requires face mask ventilation or switching to tracheal intubation			×				
Reintubation				×			
Tachycardia			×	×			
Bradycardia			×	×			
Rating of reflux degree			×				
Recovery time				×			
QoR-15 scale					×		
MoCA					×		
PONV					×		
Respiratory failure				×	×	×	×
ARDS				×	×	×	×
VAS for patients satisfaction					×		
VAS for endoscopist satisfaction					×		
Length of hospital stay							×
**Adverse Events**							
Allergy			×	×			
Headache				×	×	×	×
Epistaxis			×		×		
	**Pre-op visit**	**Pre-op**	**Perio-op**	**PACU**	**1 day post-op**	**3 day post-op**	**Discharged**
Nasal mucosal injury			×				
Oral soft tissue injury			×	×			
Tooth avulsion			×	×			
Excessive dreaming					×		
Procedure suspension due to airway compromise			×				

According to SPIRIT, statement of defining standard protocol items for clinical trials.

Pre-op, Preoperative; Perio-op, Perioperative; Post-op, Postoperative; QoR-15, patient self-assessment of Quality of Recovery-15 scale; MoCA, Montreal Cognitive Assessment; PONV, Post-operative Nausea and Vomiting; ARDS, Acute Respiratory Distress Syndrome; VAS, Visual analog scale; PACU, Post-Anesthesia Care Unit.

### 2.3 Sample size calculation

Previous studies conducted under general anesthesia have reported postoperative hypoxemia rates of approximately 10%-22.5% in the post-anesthesia care unit (PACU) (21), while the incidence of intraoperative hypotension in patients undergoing prone procedures has ranged from 20.6% to 62.5% (22, 23). Based on these findings and our pilot data, we hypothesize that ciprofol-based deep analgosedation assisted with a nasopharyngeal airway could reduce the incidence of the composite outcome (hypoxemia or hypotension) from 39% to 19%, representing an absolute risk reduction of 20%. Using a two-sided test with a significance level of α = 0.05 and 80% power, 77 participants per group are required. Allowing for potential loss to follow-up, a total of 170 patients (85 per group) will be recruited.

### 2.4 Secondary outcomes

Secondary outcomes include: Nasopharyngeal airway requires face mask ventilation or switching to tracheal intubation (Intraoperative), Reintubation after the tracheal intubation is removed (During the recovery room period); Tachycardia (>100 beats/minute) or bradycardia (< 50 beats/minute; Intraoperative and during the recovery room period); Rating of reflux degree (Intraoperative); Recovery room time (time from endoscope removal to achieving an Aldrete score of 10 and discharge from the endoscopy recovery unit; During the recovery room period); Early postoperative recovery quality (QoR-15 score) on postoperative day1 (POD 1); Postoperative nausea and vomiting (POD 1); Postoperative cognitive function assessment (Montreal Cognitive Assessment; POD 1); The incidence of perioperative respiratory failure (postoperative period); The incidence of acute respiratory distress syndrome (ARDS) during the postoperative period; Patient/endoscopist satisfaction (10-pointVAS; POD 1); Hospitalization duration (Table 1). Data will be analyzed using a modified intention-to-treat approach.

### 2.5 Randomization

This is an open-label, two-arm randomized controlled trial enrolling 170 participants allocated 1:1 to the GA or DAS group. An independent biostatistician will generate the randomization sequence using an online system (https://www.sealedenvelope.com/simple-randomiser/v1/lists), with permuted blocks of 2 and 4 and stratification by center and baseline hypertension status (yes/no). Allocation will be concealed using sealed, sequentially numbered opaque envelopes, which will be opened only after patient arrival in the operating room. Due to the inherent nature of the interventions-endotracheal intubation vs. natural-airway management with a nasopharyngeal airway-blinding of the anesthesiology and endoscopy teams is not feasible. However, postoperative outcome assessors, data managers, and statisticians will remain blinded to group allocation to reduce detection and analytical bias.

### 2.6 Anesthetic care

All patients will fast for at least 8 h and abstain from water for 4 h before the procedure. Upon arrival in the operating room, a peripheral intravenous line will be established. Continuous intraoperative monitoring will include noninvasive blood pressure, pulse oximetry, heart rate, Bispectral Index (BIS) (24), and respiratory parameters such as respiratory rate and end-tidal carbon dioxide (PETCO_2_) (25). All anesthesia and sedation procedures will be administered by experienced anesthesiologists, while ERCP will be performed by a consistent team of qualified endoscopists at each participating center. Following the procedure, all patients, regardless of group allocation, will be transferred to the endoscopy recovery area for postoperative monitoring. Continuous physiological monitoring will be conducted by anesthesiologists, who will assess recovery status and ensure that the Aldrete score reaches 10 before patients are transferred back to the ward.

NPA-assisted DAS Group, patients will be positioned in prone position. The nasal cavity will be pre-treated with lubricant and vasoconstrictive nasal spray. Preoxygenation will be performed via facemask (6 L/min), followed by intravenous administration of ciprofol (0.3–0.4 mg/kg) and sufentanil [5–10 μg (26), dosed at 0.1 μg/kg (27–29)]. After loss of consciousness, a nasopharyngeal airway will be gently inserted and connected to the anesthesia machine for low tidal volume/high-frequency oxygen delivery. Continuous infusions of ciprofol (0.8–1.2mg/kg/h) and remifentanil (0.05–0.2μg/kg/min) will be titrated based on age, weight, and clinical status to maintain stable sedation (BIS 40–60) (30). If nasopharyngeal airway placement failed in one nostril, the contralateral side was attempted. Patients will be excluded after four unsuccessful attempts or in cases of significant epistaxis.

General Anesthesia (GA) Group: Patient will be placed in supine position, anesthesia will be induced with propofol, sufentanil, and rocuronium (0.6 mg/kg) for neuromuscular blockade. After endotracheal intubation, mechanical ventilation will be initiated (tidal volume 6–8 ml/kg), FiO_2_ 40%−60%, with respiratory rate adjusted to maintain PETCO_2_ at 35–45 mmHg, then patient will be turned to the prone position. Anesthesia depth will be maintained using propofol and remifentanil as the deep sedation group with BIS-guided titration.

### 2.7 Data collection and monitoring

Data collection will encompass a range of patient characteristics, including Age, sex, ASA score, BMI, smoking status, pre-existing comorbidities, Mallampati score, interincisal distance, thyromental distance, ERCP complexity grading (14), and procedure duration (from endoscope insertion to withdrawal). Anesthesia medication dosages will be recorded. Assessors of subjective outcomes (outcomes that assessed postoperative) are blinded to group allocation. All data will be documented in case report forms (CRFs) and subsequently entered into the electronic database under the supervision of the principal investigator. An independent Data Monitoring Committee (DMC) will continuously monitor the data collection process. After all patients have completed this trial, the primary investigator and data administrator will check and confirm the integrity and accuracy of the data. Thereafter, all data will be locked. The administrator will import the data into a designated form and send it to a statistician for final statistical analyses. Any serious adverse events (SAEs), whether related or unrelated to the study (e.g., persistent hemodynamic instability or hypoxia), must be reported immediately to the principal investigator. In such cases, the perioperative care team should take the necessary measures to ensure the safety of the participants. These events must also be reported to the DMC within 24 h for further discussion and determination of whether modifications to the study interventions or termination of the study are necessary.

### 2.8 Statistical analysis

The normality of continuous variables will be assessed using the Shapiro-Wilk test. Normally distributed variables will be summarized as mean (standard deviation, SD) and compared between groups using independent-samples *t*-tests. Non-normally distributed variables will be presented as median (interquartile range, IQR) and compared using the Mann–Whitney *U* test. Categorical variables will be expressed as counts (percentages) and compared using chi-square (χ^2^) tests or Fisher's exact tests when appropriate.

The primary endpoint-the composite incidence of perioperative sedation-related adverse events (SRAEs), including hypotension, hypertension, hypoxemia, laryngospasm, or bronchospasm-will be compared between groups using χ^2^ tests. Risk estimates including odds ratios (ORs) and corresponding 95% confidence intervals (CIs) will be reported. To account for potential confounding effects, multivariable logistic regression analyses will be conducted with prespecified covariates such as age, hypertension status, ASA classification, and procedure duration, providing adjusted ORs with 95% CIs.

Secondary outcomes, including postoperative recovery quality (QoR-15) on postoperative day1, cognitive function (MoCA) on POD1, and hospital length of stay, will be analyzed using *t*-tests or Mann–Whitney *U* tests depending on distribution characteristics. Secondary categorical outcomes (e.g., postoperative nausea and vomiting, escalation of airway interventions) will be analyzed using χ^2^ tests or Fisher's exact tests as appropriate. As no multiple testing corrections are planned for secondary outcomes, these results should be interpreted as exploratory.

All primary analyses will follow a modified intention-to-treat (mITT) principle, including all randomized participants with available outcome data. A per-protocol (PP) analysis will also be conducted to evaluate robustness of results among participants without major protocol deviations. If missing data for the primary endpoint exceed 5%, sensitivity analyses using multiple imputation and inverse probability weighting methods will be performed to assess consistency of findings.

All statistical tests will be two-sided, with *P* < 0.05 considered statistically significant. Analyses will be performed using SPSS software (version 25.0; IBM, USA).

### 2.9 Patient and public involvement

Patients and the public will not participate in the study's design, recruitment, conduct, or reporting. Study results will be shared with participants via email.

## 3 Discussion

In this prospective, randomized, open-label, controlled clinical trial, we will enroll 170 elderly patients undergoing ERCP to compare perioperative sedation-related adverse events (SRAEs) between nasopharyngeal airway-assisted deep analgosedation (NPA-assisted DAS) and general anesthesia (GA). The trial will be conducted in accordance with the Consolidated Standards of Reporting Trials (CONSORT) guidelines (31). The primary outcome is the incidence of SRAEs, including hypotension, hypertension, hypoxemia, laryngospasm, and bronchospasm. Secondary outcomes include the requirement for escalation of airway interventions, tachycardia or bradycardia, degree of reflux, recovery room time, postoperative recovery quality, postoperative nausea and vomiting, postoperative cognitive function, perioperative respiratory failure, acute respiratory distress syndrome (ARDS), patient and endoscopist satisfaction, and hospital length of stay. Baseline variables will be adjusted in the analysis, and sensitivity analyses will be performed to evaluate the robustness of the findings. These methodological approaches are intended to ensure the reliability and validity of the study results. And the findings will be reported in a future publication following study completion.

Ciprofol, a novel GABA receptor agonist, provides comparable sedative efficacy at lower doses than propofol and has been associated with less respiratory and hemodynamic depression (29, 32). Previous clinical evidence in high-risk cardiac surgery patients has also indicated more stable hemodynamics with ciprofol compared with propofol (33). In this study, ciprofol is combined with continuous remifentanil (34) infusion to optimize sedation and analgesia during ERCP. In addition, nasopharyngeal airway (NPA) assistance is applied to maintain airway patency and improve oxygenation while avoiding escalation to more invasive airway management (e.g., endotracheal intubation). By simultaneously optimizing both sedative medication and airway management, our NPA-assisted deep analgosedation protocol aims to provide an efficient and safer approach that may reduce perioperative sedation-related adverse events in elderly patients undergoing ERCP.

Previous studies have reported that intraoperative hypotension (IOH) occurs in approximately 20–35% of noncardiac surgeries (7). Evidence has also shown that IOH is associated with multiple hypoperfusion-related adverse outcomes, including acute kidney injury, delirium, postoperative cognitive dysfunction, major adverse cardiac or cerebrovascular events (35), and postoperative infection (15, 36, 37). In elderly patients, a mean arterial pressure (MAP) below 65 mmHg has been associated with an increased risk of postoperative mortality (38). In addition, intraoperative hypoxemia is an important risk factor for postoperative delirium in older adults (39), and even mild oxygen desaturation during anesthesia care has been reported to elevate the likelihood of delirium within the first postoperative week. Together, these findings highlight the importance of maintaining hemodynamic stability and adequate oxygenation in elderly patients undergoing anesthesia. Moreover, the prone position used for ERCP can reduce venous return and cardiac output. In older adults with limited compensatory capacity, these circulatory changes may be more pronounced, potentially exacerbating anesthesia-related hypotension.

To standardize airway and sedation management across groups, this trial incorporates preemptive nasopharyngeal airway placement and BIS-guided titration of ciprofol-remifentanil infusion in the deep analgosedation group. In the general anesthesia group, BIS values will likewise be maintained between 40 and 60 to achieve an appropriate anesthetic depth. These measures ensure controlled and comparable sedation levels while preserving spontaneous ventilation in the deep analgosedation group. This protocolized approach helps reduce confounding effects from operator-dependent airway maneuvers and variability in sedation depth, thereby improving the internal validity of the trial.

### 3.1 Limitations

This study has several limitations. First, as an open-label trial, the lack of blinding may introduce bias, particularly in subjectively assessed secondary outcomes such as satisfaction and recovery quality. However, objective physiological parameters were selected as the primary outcomes, which helps minimize this concern. Second, follow-up is restricted to the hospitalization period, which may overlook delayed or long-term complications; future studies with extended follow-up are needed. Third, the exclusion of patients with severe chronic comorbidities may limit the generalizability of the findings to the broader ERCP population. Finally, although a composite primary endpoint was used to comprehensively assess sedation-related adverse events, the diagnostic thresholds, particularly for hypotension and hypertension, may be relatively stringent. However, strict hemodynamic control is clinically warranted in elderly patients with reduced cardiovascular reserve, supporting the relevance of these criteria in this population.

## 4 Conclusion

This randomized controlled trial will evaluate whether NPA-assisted DAS can reduce perioperative SRAEs compared with endotracheal intubation GA in elderly ERCP patients. The results are expected to provide evidence to inform anesthesia management and guide clinical decision-making in this high-risk population.
